# The first mitogenome of *Petrovinema skrjabini* from *Equus ferus przewalskii*: a phylogenetic analysis within the Strongylidae family

**DOI:** 10.1186/s13071-025-06851-7

**Published:** 2025-07-11

**Authors:** Huiping Jia, Liping Tang, Yajun Fu, Yu Xiong, Liping Yan, Changliang Shao, Kai Li, Dong Zhang, Defu Hu

**Affiliations:** 1 School of Ecology and Nature Conservation, Beijing Forestry University, Beijing, 100083 China; 2 Xinjiang Kalamaili Mountain Ungulate Nature Reserve Management Center, Xinjiang, China

**Keywords:** *Petrovinema skrjabini*, Cyathostominae, Mitochondrial genome, Phylogenetic analysis, *Equus ferus przewalskii*

## Abstract

**Background:**

*Petrovinema skrjabini* (Nematoda: Strongylidae, Cyathostominae) is a parasitic nematode colonizing the cecum and colon of equids. Like other cyathostomins, its larvae (L3) invade the intestinal mucosa, forming encysted nodules that may remain dormant for years. Mass larval emergence triggers larval cyathostominosis—a severe syndrome characterized by hemorrhagic typhlocolitis and diarrhea, with mortality rates exceeding 50%. However, owing to the morphological indistinguishability of cyathostomin and frequent mixed infections in natural settings, species-specific contributions to pathogenesis remain unresolved. Previous studies on *P. skrjabini* have predominantly focused on its morphology, with limited molecular information available.

**Methods:**

The complete mitogenome of *Petrovinema skrjabini* was sequenced using the Illumina NovaSeq 6000 platform, followed by assembly and annotation. We performed a phylogenetic analysis using Bayesian inference (BI) and maximum likelihood (ML) methods, based on 12 protein-coding genes from mitogenomes, to assess the evolutionary relationships of 34 Strongylidae species.

**Results:**

The complete mitogenome of *P. skrjabini* comprises 13,885 base pairs with 12 protein-coding genes, two ribosomal-RNA genes, 22 transfer-RNA genes, and two non-coding regions. The gene arrangement of the *P. skrjabini* mitogenome was consistent with the GA3 arrangement found in other Strongylidae species. The mitogenome exhibited a high AT bias (75.4%), which is consistent with other species in other Strongylidae species. Phylogenetic analysis showed that two *Strongylus* species (belonging to subfamily Strongylinae) formed a clade and located in the base of Strongylidae, while three *Triodontophorus* (belonging to subfamily Strongylinae) species and *P. skrjabini* formed another clade within in subfamily Cyathostominae within Strongylidae, based on 12 protein-coding genes from mitogenomes, suggesting that the genus *Triodontophorus* should transfer to the subfamily Cyathostominae.

**Conclusions:**

The characterization of the complete mitochondrial genomes of *P. skrjabini* is reported for the first time. This study provided helpful genetic markers for *P. skrjabini* identification and taxonomy, facilitating early nematode diagnosis and treatment to decrease equine parasitic nematode burdens. Our mitochondrial phylogeny analyses further corroborate the hypothesis that the genus *Triodontophorus* belongs to Cyathostominae. The present study enriches the database of strongylids mitogenomes and provides a new insight into the systematics of the family Strongylidae.

**Graphical Abstract:**

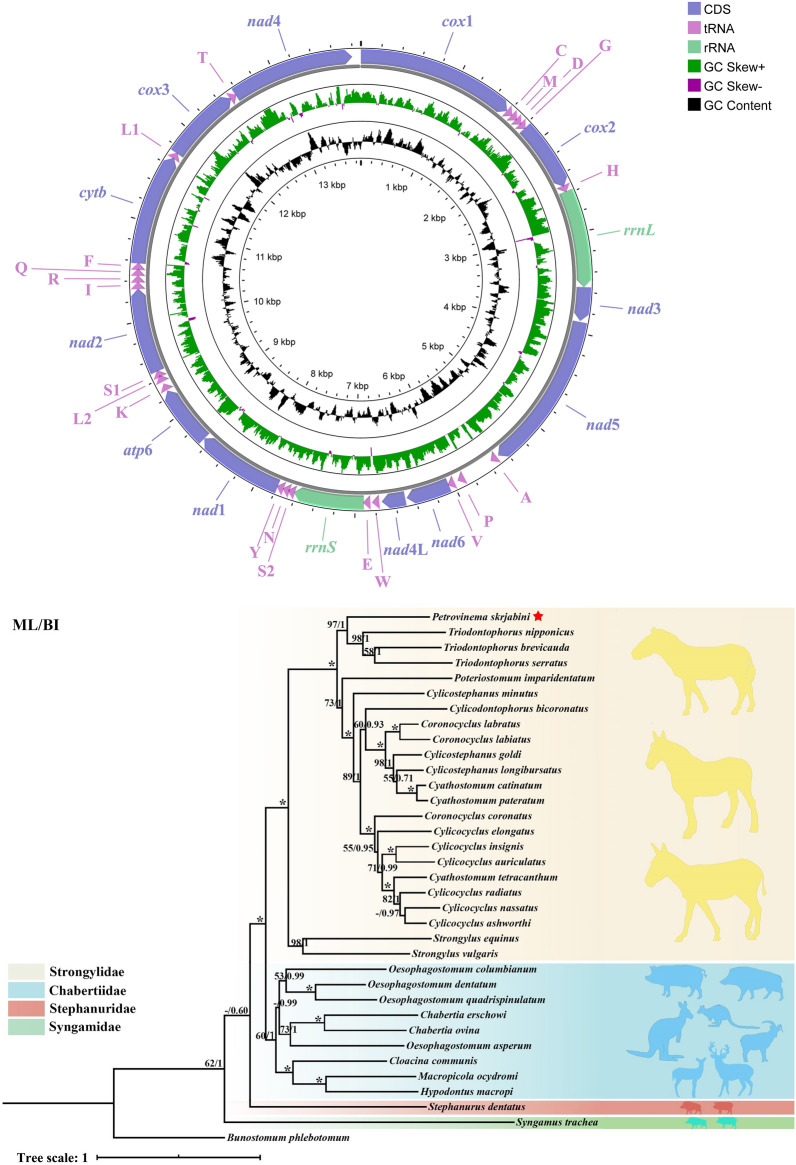

## Background

Strongylids (Nematoda: Strongylidae) are a group of parasitic nematodes that co-infect the gastrointestinal tracts of equids [1–3], with veterinary and economic importance for domestic animals and wildlife [4–10]. Co-infecting nematode species can interact within the host, changing infection dynamics and transmission, with implications for host diseases [11–14]. Hence, a detailed understanding of strongylid species and how they infect the host is essential.

Studies on strongylid communities in equids rely heavily upon the morphological identification of specimens collected from feces or culled animals [15]. This method is challenging and labor-intensive, limiting the number of hosts that can be examined and requiring considerable expertise [15, 16]. Traditionally, Strongylidae is divided into two subfamilies, Strongylinae (large strongyles with globular or funnel-shaped buccal capsules) and Cyathostominae (small strongyles with cylindrical buccal capsules), based solely on morphological characteristics [22]. However, molecular studies are inconsistent with morphological classification criteria [19, 25–27]. For instance, Gao et al. showed through mitochondrial genome analyses that the genus *Triodontophorus*, morphologically assigned to Strongylinae, cluster within Cyathostominae in mitochondrial phylogenies [30], demonstrating the limitations of relying solely on buccal capsule morphology. These findings highlight the need for molecular approaches to reconcile taxonomic uncertainties. Advances in molecular technologies open promising avenues for nematode research, providing tools for species identification, exploring molecular evolution, and conducting phylogenetic studies [17–21]. To date, approximately 64 species from 19 genera in the Strongylidae family have been described [22]; however, only 22 compete mitogenomes belonging to 8 genera within Strongylidae are available in the GenBank Database (https://www.ncbi.nlm.gov/; accessed on 20 November 2023) [23, 24].

*Petrovinema*, described by Ershov in 1943, belongs to the phylum Nematoda, family Strongylidae, and subfamily Cyathostominae. It primarily infects equids such as donkeys and horses. Infection occurs via the fecal–oral route, causing symptoms such as subcutaneous edema, diarrhea, fever, and emaciation, with approximately 50% mortality [4, 28]. In 1975, Lichtenfels classified two species of *Petrovinema*, *P. skrjabini* and *P. poculatum*, into the genus *Cylicostephanus*. In 1986, Hartwich suggested that two species of *Petrovinema* transferred to the genus of *Cylicostephanus* is unreasonable based on morphological characteristics [22], which supported using the ITS 2 sequence by Hung et al. and recognized *Petrovinema* as an independent genus [23]. Currently two species of the genus *Petrovinema* are accepted: *P. skrjabini* and *P. poculatum*. In comparison with that of *P. poculatum*, the distribution range of *P. skrjabini* is relatively limited, has only been reported in Asia [22, 29], and lacks molecular data, which, to a certain extent, hinders the attainment of a comprehensive understanding of this strongylid species. Advanced studies of the genus *Petrovinema* have mainly focused on morphological evidences, while only a small number of studies have addressed their phylogenetic relationships based on molecular sequences, especially those including mitogenomes [22].

Therefore, this study addresses this critical gap by determining the complete mitogenome of *P. skrjabini,*, which will enable a more accurate identification of the species, and thus help to determine the specific parasite type of infection. Moreover, we sought to resolve taxonomic controversies related to the Strongylidae family.

## Methods

### Sample collection and DNA extraction

*Petrovinema skrjabini* specimens were collected from *Equus ferus przewalskii* dewormed with ivermectin in the Kalamaili Nature Reserve (Xinjiang, China). The specimens were thoroughly washed with saline solution, fixed in 70% ethanol, and stored at −40 °C. Nematodes were identified using a compound microscope (Olympus CX22, Japan), with reference to taxonomic keys and descriptions [22]. A tissue sample was taken from an adult *P. skrjabini* specimen for DNA extraction. The remaining body parts of the specimen were preserved as vouchers, labeled with the number 418–6, and then deposited in the Museum of Beijing Forestry University (Ne-418). DNA was extracted using the QIAamp DNA Blood Mini Kit, following the manufacturer’s instructions (Qiagen, GmbH, Germany), and eluted from the column using 200 μL of deionized water.

### DNA annotation, sequence analysis, and comparative analysis

To survey the *P. skrjabini* genome, we generated TruSeq libraries using the Illumina NovaSeq 6000 platform (with 150 base pair [bp] paired-end reads) at Berry Genomics in China. Trimmomatic v0.38 [30] was used to eliminate highly redundant and low-quality sequences from the FASTQ data generated using the Illumina platform. De novo assemblies were created using IDBA-UD (http://i.cs.hku.hk/~alse/hkubrg/projects/idba_ud/) with a similarity threshold of 98% and *k*-values ranging from 80 to 124 bp considered. The *COXI* gene (GenBank: KP693427.1) sequences of close relatives (*P. poculatum*) served as “bait” references for obtaining the most appropriate and targeted mitochondrial scaffolds. The *P. skrjabini* mitogenome was annotated using the MITOS webserver [31], and mitochondrial DNA maps were generated utilizing the Proksee Server (https://proksee.ca/).

Protein-coding genes (PCGs) and ribosomal-RNA (rRNA) genes were compared with homologous genes from other strongylid nematodes using MEGA version 6 [32] (Table 1). The AT/GC skew, a metric for assessing strand bias, was computed as described by Perna and Kocher (1995) [33]. To analyze codon usage, the relative synonymous codon usage (RSCU) values of 12 PCGs (with their termination codons removed) were calculated using MEGA v6 [34]. The synonymous (Ks) and non-synonymous (Ka) substitution rates of the PCGs were computed using DnaSP version 5 [35]. We sorted the output data in terms of codon usage and counts and created graphics using the ggplot2 package implemented in R v3.4.1 [36]. Pairwise alignments of the 12 PCGs were conducted using MEGA v6 [32] to identify variable nucleotide sites. Sequence variability across the Strongylidae family, encompassing 22 species (including *P. skrjabini*), was assessed through sliding-window analysis using DnaSP v5 [37]. The sliding-window analysis was carried out as previously described [38]. To evaluate substitution saturation at each codon position in the PCGs, we employed Xia’s test [39] within the DAMBE.

**Table 1:** Species used for reconstructing relationships in the present study

Superfamily	Family	Subfamily	Species	Locus	Sequence length (bp)
Strongyloidea	Strongylidae	Cyathostominae	*Cylicodontophorus bicoronatus*	NC_042141	13,756
			*Coronocyclus coronatus*	OR834861.1	13,870
			*Coronocyclus labiatus*	NC_042234	13,827
			*Coronocyclus labratus*	MN832736	13,856
			*Cylicocyclus radiatus*	NC_039643	13,836
			*Cylicocyclus ashworthi*	NC_046711	13,876
			*Cylicocyclus auriculatus*	NC_043849	13,831
			*Cylicocyclus elongatus*	NC_060748	13,875
			*Cylicocyclus insignis*	NC_013808	13,828
			*Cylicocyclus nassatus*	NC_032299	13,846
			*Cyathostomum catinatum*	NC_035003	13,838
			*Cyathostomum pateratum*	NC_038070	13,822
			*Cyathostomum tetracanthum*	MN792800	13,839
			*Cylicostephanus goldi*	AP017681	13,827
			*Cylicostephanus longibursatus*	NC_081015.1	13,807
			*Cylicostephanus minutus*	NC_035004	13,826
			*Poteriostomum imparidentatum*	NC_035005	13,817
			*Strongylus equinus*	NC_026868	14,545
			*Strongylus vulgaris*	GQ888717	14,301
			*Triodontophorus brevicauda*	NC_026729	14,305
			*Triodontophorus nipponicus*	NC 031517	13,701
			*Triodontophorus serratus*	KX185154	13,794
	Chabertiidae	Chabertiinae	*Chabertia erschowi*	KF660603	13,705
			*Chabertia ovina*	NC_013831	13,682
		Cloacininae	*Cloacina communis*	NC_067626	13,689
		Oesophagostominae	*Oesophagostomum asperum*	NC_023932	13,672
			*Oesophagostomum columbianum*	NC_023933	13,561
			*Oesophagostomum dentatum*	GQ888716	13,869
			*Oesophagostomum quadrispinulatum*	NC_014181	13,681
		Phascolostrongylinae	*Hypodontus macropi*	NC_023098	13,634
			*Macropicola ocydromi*	NC_023099	13,659
	Syngamidae	–	*Syngamus trachea*	NC_013821	14,647
	Stephanuridae	–	*Stephanurus dentatus*	MW970029	13,735
Ancylostomatoidea	Ancylostomatidae	Bunostominae	*Bunostomum phlebotomum* (outgroup)	NC_012308	13,790

### Phylogenetic analyses

Phylogenetic analyses were performed using 12 PCG matrixes from the mitogenome of *P. skrjabini* (this study) and 33 other Strongyloidea mitogenomes (obtained from the NCBI GenBank database; Table 1), with *Bunostomum phlebotomum* selected as the outgroup species [40]. Maximum likelihood (ML) and Bayesian inference (BI) analyses were performed using RAxML v8 [41] and MrBayes v3.2.7a [42] as described by Zhao et al. (2021, 2022) [43, 44]. The best substitution models for the 12 PCG sequences were determined using ModelTest-NG software v0.1.7 [45]. The general time-reversible (GTR) + invariable sites (*I*) + gamma distribution (*G*) substitution model was used for the ML analysis, involving 1000 bootstrap replications. For the BI analysis, two independent runs of four Markov chains (three heated, one cold) were conducted for two million generations, sampling every 1000 generations. The GTR + *I* + *G* substitution model was used. The first 25% of the sampled trees were discarded as burn-in. Convergence of the MCMC chains was assessed by monitoring the average standard deviation of split frequencies, which fell below 0.01. The phylogenetic trees were visualized using the iTOL online tool (https://itol.embl.de) [46].

## Results

### Composition and organization of the mitogenome

**Fig. 1:**
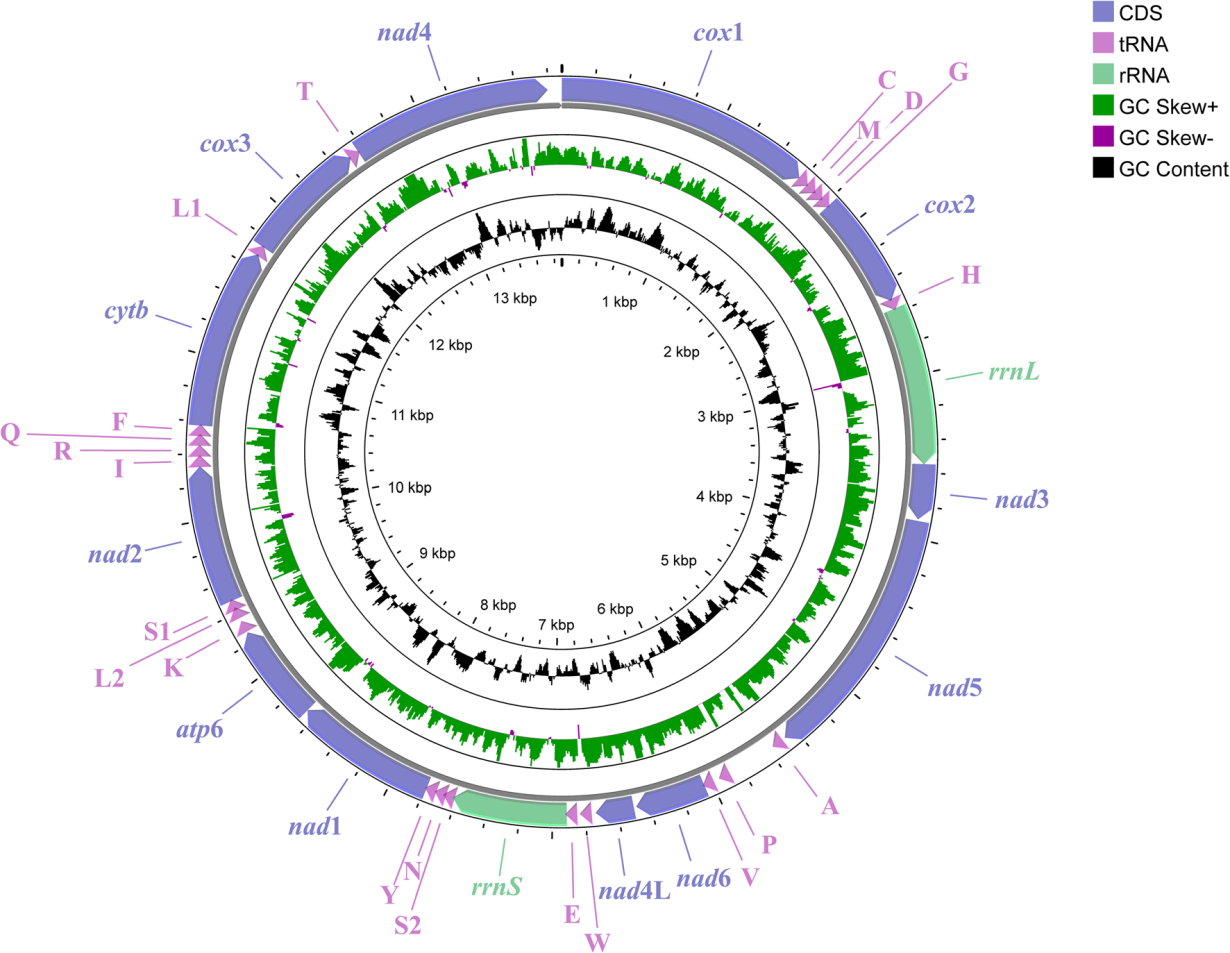
Organization of the complete mitochondrial genome of *Petrovinema skrjabini*

**Fig. 2:**
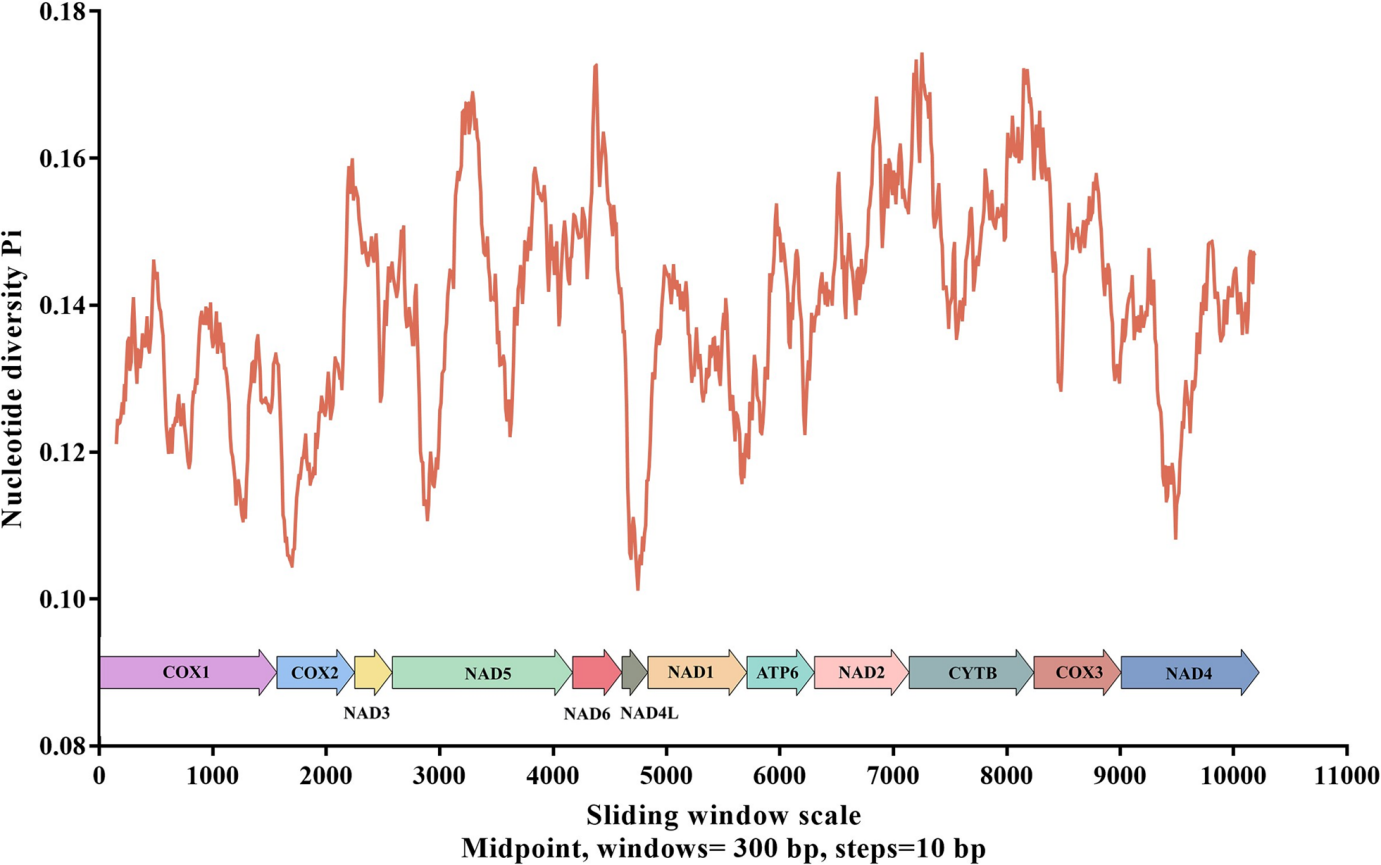
A + T content and nucleotide skew of genes, individual elements, and the complete mitogenome of *Petrovinema skrjabini*

**Fig. 3:**
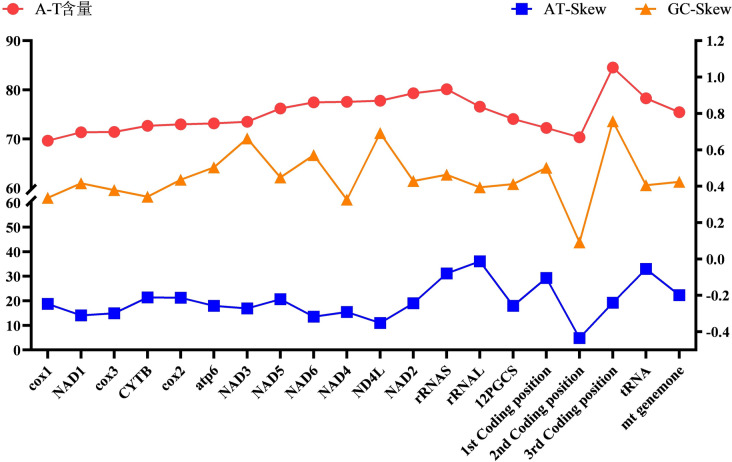
Sliding window analysis (window size 300 bp, step size 10 bp) of the alignment of mitochondrial protein-coding sequences of *Petrovinema skrjabini* used to estimate nucleotide diversity Pi (π) across the alignments. Nucleotide diversity was plotted against the mid-point positions of each window. Gene boundaries are indicated above the graph

The complete mitogenome of *P. skrjabini* spanned 13,885 bp in length and contained 36 genes, including *atp6*, *cox1*–*cox3*, *cytb*, *nad1*–*nad6*, and *nad4L*, as well as the small and large subunit rRNA genes (rrnS and rrnL), 22 tRNA genes (*tRNA-Ala*, *tRNA-Cys*, and *tRNA-Met*, etc.) and two non-coding control regions (NCRs) (Fig. 1; Table 2), and has been deposited in the NCBI database (GenBank accession number: OP784481). All genes were encoded by the same strand, and the gene arrangement (GA) conformed to GA3 [47]. Notably, the *P. skrjabini* mitogenome contained 16 intergenic spacers, ranging from 1 to 54 bp in length, and five overlaps, reflecting a highly compact organization (Table 2). The overall AT content in the *P. skrjabini* mitogenome was 75.4%, with an AT skew of −0.20 and a CG skew of 0.42 (Fig. 2). Sliding-window analysis across the 12 PCGs revealed that *nad4L* had the lowest nucleotide variability, while *cytb* showed the highest (Fig. 3). Among the most conserved genes were *nad4L*, *cox2*, and *nad4*; whereas, *cytb*, *cox3*, and *nad6* were the least conserved.

**Table 2:** Organization of the complete mitochondrial genome of *Petrovinema skrjabini*

Genes/regions	Positions and sequence lengths (bp)	Intergenic nucleotides	Initiation/stop codons
*cox1*	1–1578 (1578)	0	ATT/TAA
tRNA-Cys(tgc)	1585–1640 (56)	6	
tRNA-Met(atg)	1648–1706 (59)	7	
tRNA-Asp(gac)	1708–1765 (58)	1	
tRNA-Gly(gga)	1770–1824 (55)	4	
*cox2*	1825–2520 (696)	0	ATT/TAA
tRNA-His(cac)	2520–2573 (54)	−1	
*rrnL*	2572–3547 (974)	−2	
*nad3*	3548–3883 (336)	0	ATT/TAA
*nad5*	3895–5478 (1584)	11	ATT/TAA
tRNA-Ala(gca)	5508–5562 (55)	29	
LNCR	5563–5889 (326)	0	
tRNA-Pro(cca)	5890–5943 (54)	0	
tRNA-Ser(gta)	5998–6051 (54)	54	
*nad6*	6052–6486 (435)	0	ATT/TAG
*nad4L*	6503–6736 (234)	33	ATT/TAG
tRNA-Trp(tga)	6770–6826 (57)	31	
tRNA-Glu(gaa)	6858–6915 (58)	−1	
*rrnS*	6915–7618 (704)	−1	
tRNA-Ser2(tca)	7618–7670 (53)	0	
tRNA-Asn(aac)	7671–7727 (57)	7	
tRNA-Tyr(tac)	7735–7789 (55)	0	
*nad1*	7790–8662 (873)	1	TTG/TAG
*atp6*	8664–9263 (600)	12	ATT/TAA
tRNA-Lys(aaa)	9276–9338 (63)	34	
tRNA-Leu2(tta)	9373–9427 (55)	0	
tRNA-Ser1(aga)	9428–9479 (52)	0	
*nad2*	9480–10325 (846)	2	TTG/TAG
tRNA-Ile(atc)	10,328–10,387 (60)	6	
tRNA-Arg(cgt)	10,394–10,449 (56)	11	
tRNA-Gln(caa)	10,461–10,515 (55)	3	
tRNA-Phe(ttc)	10,519–10,575 (57)	0	
*cytb*	10,576–11688 (1113)	−1	ATT/TAA
tRNA-Leu1(cta)	11,688–11,743 (56)	0	
*cox3*	11,744–12,509 (766)	0	ATT/T
tRNA-Thr(aca)	12,510–12,568 (59)	0	
*nad4*	12,569–13,798 (1230)	0	TTG/TAA
SNCR	13,799–13,885 (87)	0

### PCGs and codon usage

**Fig. 4:**
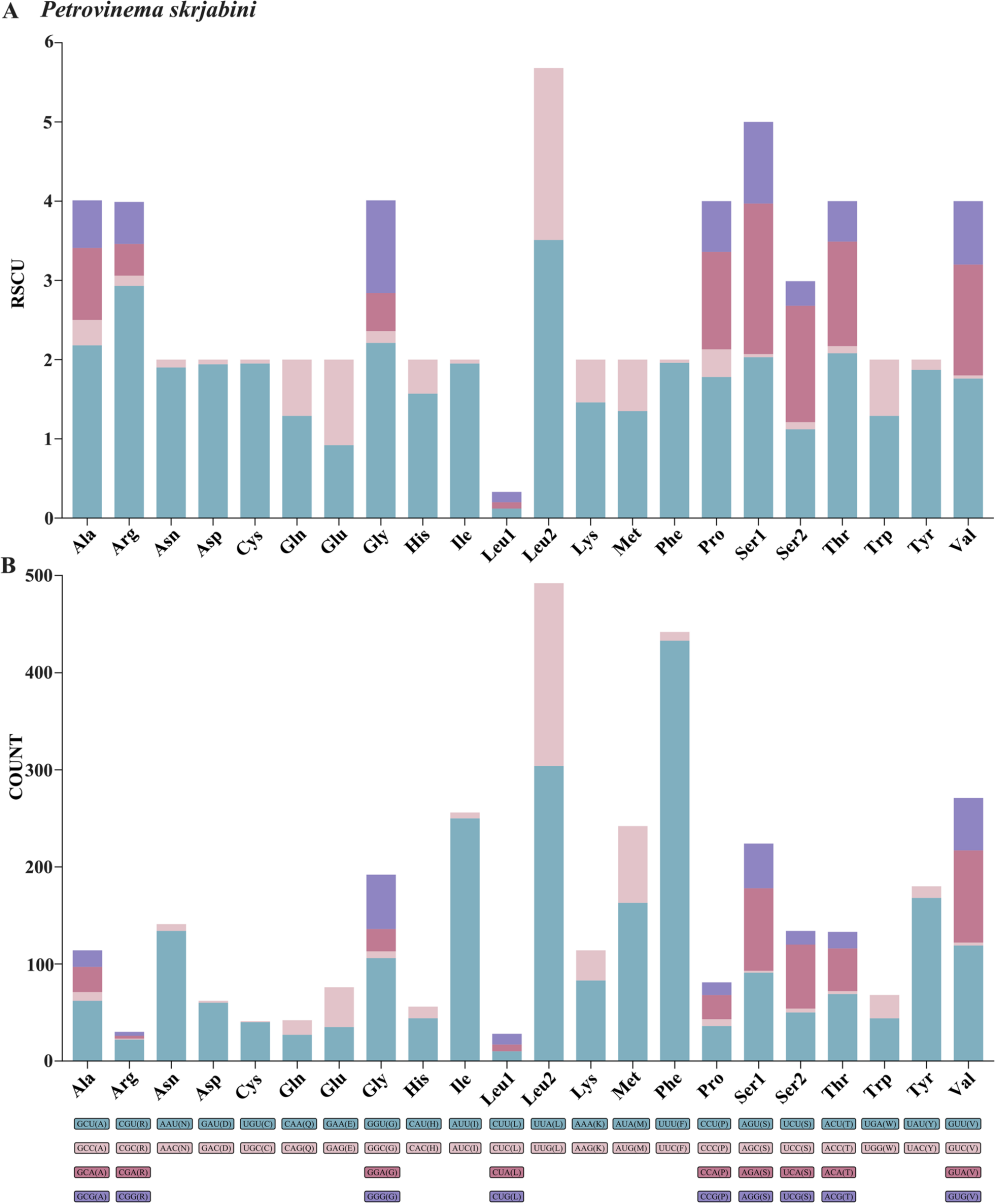
Relative synonymous codon usage (RSCU, **A**) and COUNT (**B**) of *Petrovinema skrjabini* mitogenome

A total length of 10,291 bp was assembled across the 12 PCGs of *P. skrjabini*, encoding 3419 amino acids (Table 3). The results showed that T was the most prevalent base in all 12 PCGs of *P. skrjabini*, followed by G, A, and C. The AT contents of the 12 PCGs ranged from 69.65% (*cox1*) to 79.31% (*nad2*), with the third codon positions exhibiting the highest AT content (84.55%) compared with the first (72.25%) and second positions (70.35%). The high usage frequencies of codons such as UUU (Phe) and UUA (Leu) contributed to the overall high AT content of the mitogenome, whereas codons such as CUC (Leu) and GUC (Val) were rarely used (Fig. 4A and B).

**Table 3:** Lengths and amino acids of mitochondrial genes/regions of 17 Strongylidae species

	*Pet. skrjabini*	*Cya. catinatum*	*Cya. pateratum*	*Cya. tetrac* *anthum*	*Cylicos. goldi*	*Cylicos. minutus*	*Cor. labiatus*	*Cor. labratus*	*Cylicoc. auriculatus*	*Cylicoc. insignis*	*Cylicoc. elongatus*	*Cylicoc. nassatus*	*Cylicoc. radiatus*	*Cylicoc. ashworthi*	*Cylicod. bicoronatus*	*Pot. imparidentatum*	*Str. equinus*	*Str. vulgaris*	*Tri. brevicauda*	*Tri. nipponicus*	*Tri. serratus*
*cox1*	1578/525	1578/525	1575/524	1578/525	1578/525	1578/525	1578/525	1578/525	1578/525	1578/525	1578/525	1578/525	1578/525	1578/525	1578/525	1578/525	1578/525	1578/525	1578/525	1578/525	1578/525
*cox2*	696/231	696/231	696/231	696/231	696/231	696/231	696/231	696/231	696/231	696/231	696/231	696/231	696/231	696/231	696/231	696/231	696/231	697/231	696/231	696/231	696/231
*rrnL*	974/–	976/–	976/–	977/–	972/–	972/–	979/–	975/–	969/–	959/–	968/–	974/–	969/–	978/–	982/–	983/–	959/–	959/–	975/–	976/–	961/–
*nad3*	336/111	336/111	336/111	336/111	336/111	336/111	336/111	336/111	336/111	336/111	336/111	336/111	336/111	336/111	336/111	336/111	336/111	336/111	336/111	336/111	336/111
*nad5*	1584/527	1584/527	1584/527	1584/527	1593/530	1584/527	1584/527	1584/527	1584/527	1584/527	1584/527	1584/527	1584/527	1584/527	1584/527	1584/527	1599/532	1584/527	1584/527	1584/527	1584/527
*nad6*	435/144	435/144	435/144	435/144	435/144	435/144	435/144	435/144	435/144	435/144	435/144	435/144	435/144	435/144	438/145	435/144	435/144	435/144	435/144	435/144	435/144
*nad4L*	234/77	234/77	234/77	234/77	234/77	234/77	234/77	234/77	234/77	234/77	234/77	234/77	234/77	234/77	234/77	234/77	234/77	234/77	234/77	234/77	234/77
*rrnS*	704/–	708/–	698/–	700/–	699/–	700/–	701/–	701/–	699/–	700/–	699/–	699/–	697/–	711/–	702/–	709/–	708/–	700/–	703/–	696/–	701
*nad1*	873/290	873/290	873/290	873/290	873/290	873/290	873/290	873/290	873/290	873/290	873/290	873/290	873/290	873/290	873/290	873/290	879/292	876/291	873/290	873/290	873/290
*atp6*	600/199	600/199	600/199	600/199	600/199	600/199	600/199	600/199	600/199	600/199	600/199	600/199	600/199	600/199	600/199	600/199	600/199	603/200	600/199	600/199	600/199
*nad2*	846/281	846/281	846/281	846/281	846/281	846/281	846/281	846/281	846/281	846/281	846/281	846/281	846/281	846/281	846/281	846/281	846/281	846/281	846/281	846/281	846/281
*cytb*	1113/370	1113/370	1113/370	1113/370	1113/370	1113/370	1110/369	1113/370	1113/370	1113/370	1113/370	1113/370	1113/370	1113/370	1113/370	1113/370	1113/370	1116/371	1113/370	1113/370	1113/370
*cox3*	766/255	766/255	766/255	766/255	766/255	766/255	766/255	766/255	766/255	769/256	766/255	766/255	766/255	766/255	766/255	766/255	766/255	766/255	766/255	766/255	766/255
*nad4*	1230/409	1230/409	1230/409	1230/409	1230/409	1230/409	1230/409	1230/409	1230/409	1230/409	1230/409	1230/409	1230/409	1230/409	1230/409	1227/408	1230/409	1230/409	1230/409	1230/409	1230/409
Total	11,967	11,975	11,962	11,968	11,971	11,963	11,968	11,967	11,959	11,953	11,958	11,964	11,957	11,980	11,978	11,980	11,979	11,960	11,969	11,963	11,953

*Pet. skrjabini, Petrovinema skrjabini; Cya. Catinatum, Cyathostomum catinatum; Cya. pateratum, Cyathostomum pateratum; Cya. Tetracanthum, Cyathostomum tetracanthum; Cylicos. goldi, Cylicostephanus goldi; Cylicos. minutus, Cylicostephanus minutus; Cor. labiatus, Coronocyclus labiatus; Cor. labratus, Coronocyclus labratus; Cylicoc. auriculatus, Cylicocyclus auriculatus; Cylicoc. insignis, Cylicocyclus insignis; Cylicoc. elongatus, Cylicocyclus elongatus; Cylicoc. nassatus, Cylicocyclus nassatus; Cylicoc. radiatus, Cylicocyclus radiatus; Cylicoc. ashworthi, Cylicocyclus ashworthi; Cylicod. bicoronatus, Cylicodontophorus bicoronatus; Pot. imparidentatum, Poteriostomum imparidentatum; Str. equinus, Strongylus equinus; Str. vulgaris, Strongylus vulgaris; Tri. brevicauda, Triodontophorus brevicauda; Tri. nipponicus, Triodontophorus nipponicus; Tri. serratus, Triodontophorus serratus*

### tRNA genes, rRNA genes, and non-coding regions

The *P. skrjabini* mitogenome contained 22 tRNA genes, ranging in length from 52 to 63 bp, these tRNAs appeared to fold into an atypical clover-leaf secondary structure. Two tRNAs, namely *tRNA-Ser2* and *tRNA-Ser1*, lacked a dihydrouridine arm and had a d-loop structure. The other 20 tRNAs lacked the TΨC loop, which was replaced by the “TV-replacement loop”. The two rRNA genes, rrnL (974 bp) and rrnS (704 bp), were located between *tRNA-His* and *nad3*, and between *tRNA-Glu* and *tRNA-Ser*, respectively. The AT contents of rrnL and rrnS were 80.1% and 76.6%, respectively. Two NCRs were identified: the long NCR (326 bp) located between *tRNA-Ala* and *tRNA-Pro*, and the short NCR (87 bp) located between *nad4* and *cox1* (Table 2), with AT contents of 87.8% and 80.5%, respectively. The rrnL gene was located between *tRNA-His* and *nad3*, whereas rrnS was located between *tRNA-Glu* and *tRNA-Ser*.

### Phylogenetic analyses

**Fig. 5:**
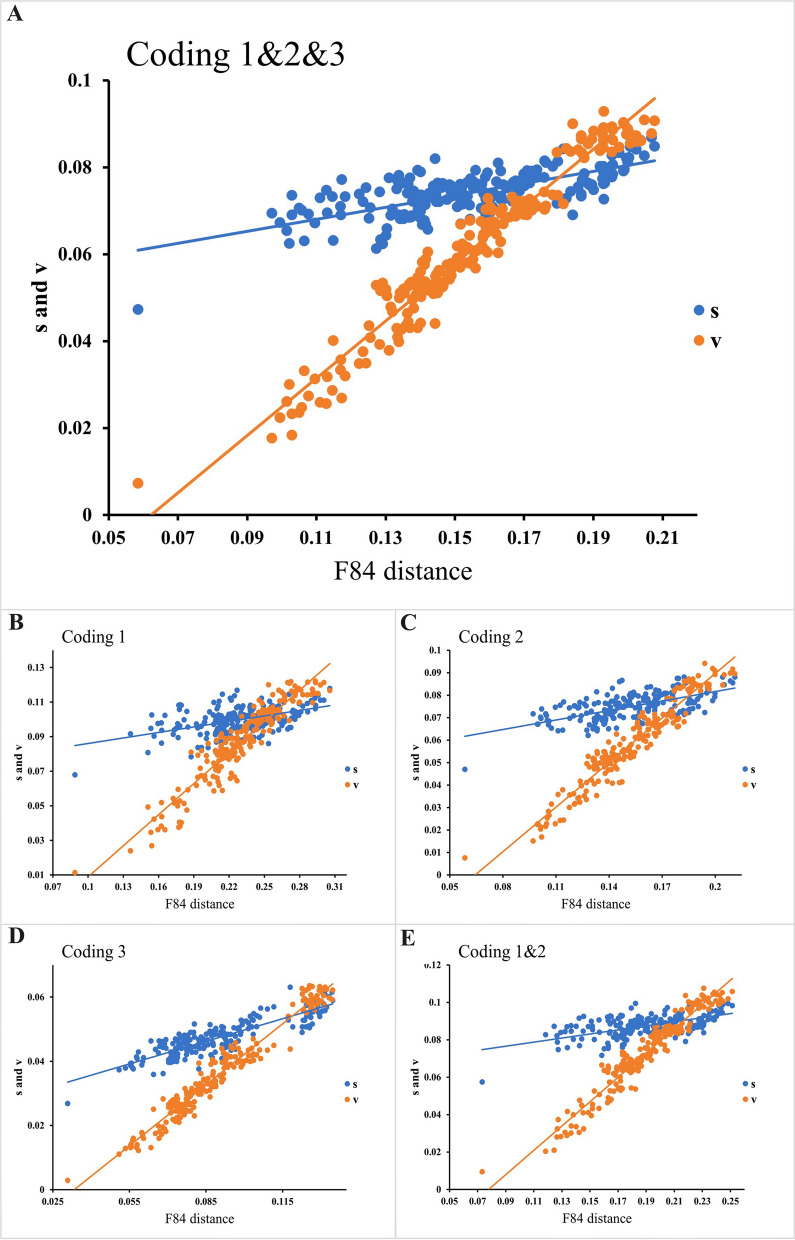
The transitions (s) and transversions (v) for 12 protein-coding genes of Strongyloidea against GTR distance. Plots in blue and orange indicate transition and transversion, respectively. (**A**) Coding genes 1, 2 and 3; (**B**) Coding gene 1; (**C**) Coding gene 2; (**D**) Coding gene 3; (**E**) Coding genes 1 and 2

**Fig. 6:**
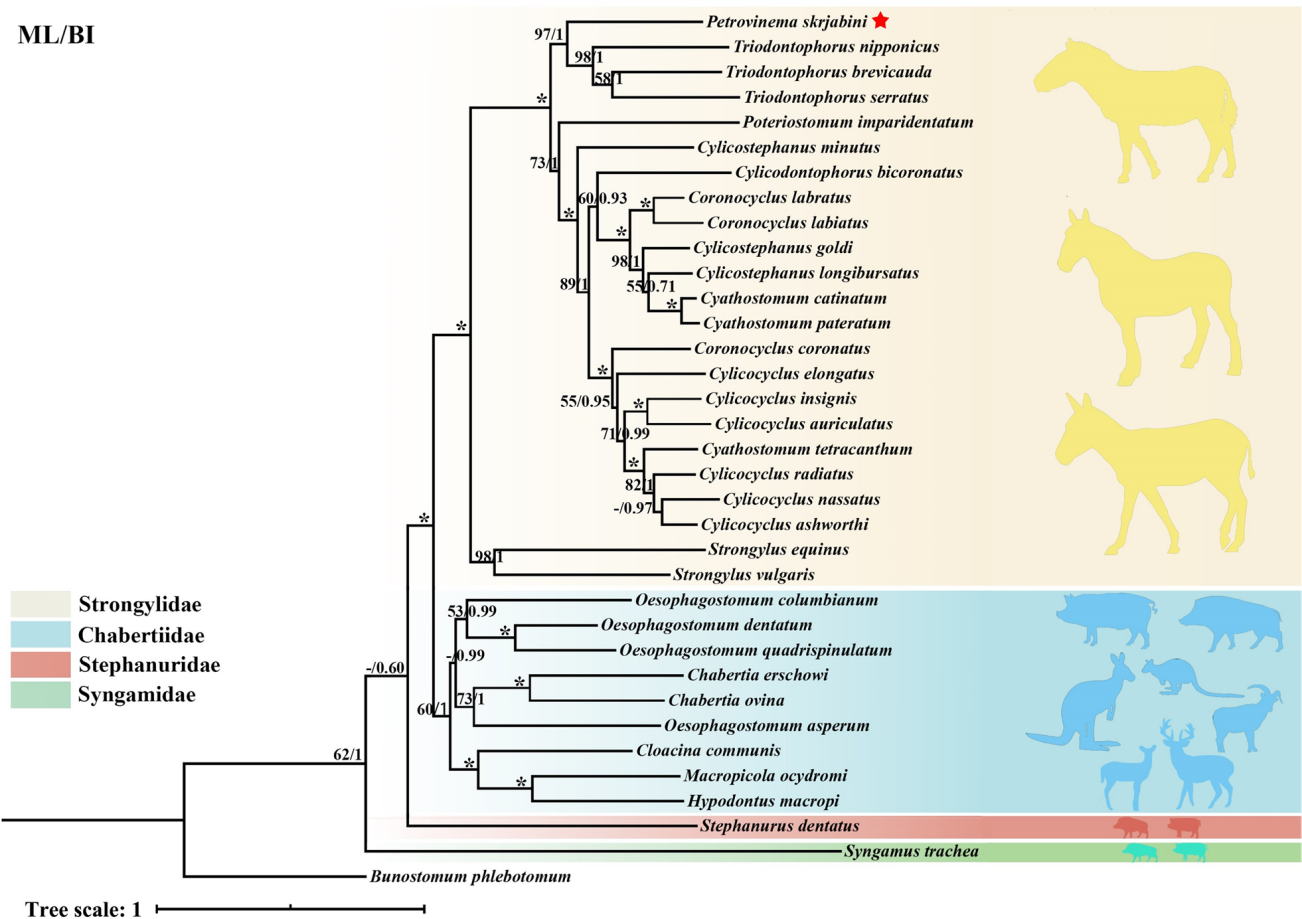
Phylogenetic relationships of Strongyloidea inferred from mitochondrial genomes. Topology obtained on the basis of concatenated amino acid sequences of 12 protein coding genes analyzed by maximum likelihood (ML) and Bayesian inference (BI) using *Bunostomum phlebotomum* as an outgroup. Statistical support values (bootstrap/posterior probability) of ML/BI analysis are shown above the nodes. Asterisks (*) indicate ML/BI = 100/1.0, other values are given above the nodes. Families are highlighted by individual colors. The species identified in the present study is denoted by the red star. Values below 50% are shown here as “–”

Prior to phylogenetic reconstruction, substitution saturation analysis of the 12 PCGs was performed (Fig. 5). The substitution saturation index (Iss) was significantly lower than the critical Iss value (P < 0.001), confirming minimal saturation and validating the suitability of these sequences for phylogenetic inference. Phylogenetic analyses were reconstructed using concatenated sequences of the 12 PCGs from *P. skrjabini* and 33 other Strongyloidea species representing 16 genera (data from GenBank). The maximum likelihood (ML) and Bayesian inference (BI) analyses produced identical tree topologies (Fig. 6). While most nodes were well-supported, some nodes in Oesophagostominae and Strongylidae displayed weak bootstrap support (< 50) in ML but were strongly supported in BI (posterior probability ≥ 0.97). The Cyathostominae subfamily formed a strongly supported monophyletic clade (bootstrap = 100; posterior probability = 1), including genera *Cylicocyclus*, *Cyathostomum*, *Coronocyclus*, *Poteriostomum*, *Cylicostephanus*, *Cylicodontophorus*, *Petrovinema*, and *Triodontophorus* (traditionally classified under Strongylinae). In contrast, Strongylinae was paraphyletic, with *Strongylus vulgaris* and *S. equinus* forming a basal clade, while *Triodontophorus* clustered within Cyathostominae. *Petrovinema skrjabini* clustered robustly with three species of the *Triodontophorus* genus (posterior probability = 1; bootstrap = 97), forming a monophyletic group that further clustered with six other genera within the Cyathostominae subfamily (Fig. 6).

## Discussion

Previous studies on the *Strongyloidea* superfamily have primarily relied on single-gene markers or morphological characteristics, which often lack the resolution required to resolve complex evolutionary relationships, particularly for the genus *Petrovinema*. In this study, we sequenced and analyzed the complete mitochondrial genome of *P. skrjabini*, revealing its gene content, arrangement, and codon usage. This work not only expands the available genomic resources for Strongyloidea nematodes but also offers new insights into their evolutionary relationships and phylogenetic placement.

The gene arrangement of *P. skrjabini* conformed to the GA3 [47] pattern, consistent with other strongylids such as *T. brevicauda*, *Strongylus equinus*, *Cylicocyclus insigne*, and *Cylicodontophorus bicoronatus* [23, 24, 48–52]. This structural conservation reflects evolutionary stability and strong selective pressures within the Strongyloidea superfamily. The absence of the *ATP8* gene in *P. skrjabini* and other Strongyloidea species, is likely due to functional redundancy or gene loss during evolution. The notable AT bias in the *P. skrjabini* mitogenome (75.4%) was consistent with Strongyloidea mitogenomes [24, 52–54] (Fig. 2), with NCR AT content (86.5%) similar to those observed in nematodes and trematodes, such as *Cylicocyclus radiatus*, *Cyathostomum catinatum*, *Coronocyclus labiatus*, *Echinostoma miyagawai*, and *Postharmostomum commutatum* [24, 55, 56]. In addition, the size and location of NCRs in the *P. skrjabini* mitogenome resembled those in the subfamily of Cyathostominae nematodes (Table 3) but differed from those of the subfamily of Strongylinae nematodes [48–50]. Regarding nucleotide composition, the highest AT-skew value was found at the third codon position, and the lowest in the second codon position, consistent with observations in nematodes and trematodes, such as *Cylicocyclus radiatus*, *Coronocyclus labiatus*, *Cylicodontophorus bicoronatus*, and *E. miyagawai* [23, 24, 55]. These findings suggest that the nucleotide composition and skewness in the *P. skrjabini* mitogenome are consistent with evolutionary patterns observed in related species, indicating conserved mutational pressures and selection mechanisms across different nematode and trematode taxa.

The mitochondrial codon usage in *P. skrjabini* and related Strongylidae species follows the standard invertebrate genetic code, with UUA (Leu) and CGU (Arg) being the most common, consistent with 22 published Strongylidae mitogenomes [23, 24, 50, 55]. Minor interspecific differences in RSCU values were observed: CUC (Leu) has an RSCU of 0.01 in *Cylicodontophorus bicoronatus* but was absent (RSCU = 0) in *P. skrjabini* and *Coronocyclus labiatus*. Similarly, CGC (Arg) showed limited usage (RSCU = 0.13) in *P. skrjabini* but was absent (RSCU = 0) in *Cylicocyclus radiatus* and *Cylicodontophorus bicoronatus* [23, 24]. These RSCU differences highlight evolutionary divergence in codon preference, not alterations to the genetic code, aligning with the conserved framework reported for Strongylidae nematodes [23, 24, 50, 52]. The rRNA genes, rrnL and rrnS, in *P. skrjabini* are of similar length to those in other nematodes, such as *T. brevicauda* (975 bp and 703 bp), *Po. imparidentatum*, *Chabertia erschowi* (970 bp and 696 bp), and *Chabertia ovina* (962 bp and 696 bp) [24, 34, 48, 50, 57, 58]. There was no difference compared with other Strongylinae nematodes, such as *Cylicocyclus radiates* [57], in the position of rrnS. The rrnL gene was located between *tRNA-His* and *nad3*; whereas, rrnS was located between *tRNA-Glu* and *tRNA-Ser*.

This study revealed the phylogenetic relationships of the genus *Petrovinema* through mitogenome analysis of *P. skrjabini*. Contrary to the findings of Bu et al. (2016), based on *P. poculatum* ITS1/ITS2 sequence data [59], which suggested a close phylogenetic relationship between *Petrovinema* and *Poteriostomum*, our mitochondrial genomic data demonstrate that *P. skrjabini* forms a monophyletic clade with *Triodontophorus* (bootstrap/posterior probability = 97/1). This difference could potentially be due to differences in gene fragment selection and length used for phylogenetic tree construction, as longer mitochondrial genome segments provide better resolution for evolutionary trees. Owing to its relatively long length, the mitochondrial genome contains more informative data and more accurately represents the evolutionary relationships among parasitic nematodes. Similarly, the taxonomic discordance between the mitochondrial clade of *Petrovinema* and the nuclear gene clade of *Poteriostomum* underscores the necessity of integrating multi-genomic datasets in resolving phylogenies of morphologically convergent groups.

The mitochondrial phylogenetic position of the genus *Triodontophorus* (nested within the subfamily Cyathostominae), exhibits a clear inconsistency with its morphological classification under Strongylinae (Fig. 6). This incongruence corroborates the conclusions of Hung et al. and Gao et al. [27, 60]. Our results further validate that the traditional classification, dividing Strongylidae into the Strongylinae and Cyathostominae subfamilies [22] based solely on the size and shape of the buccal capsule is insufficient. On the basis of the existing molecular data, we propose transferring *Triodontophorus* to Cyathostominae while retaining Strongylinae as a monotypic subfamily containing the genus *Strongylus*. The phylogenetic analysis in this study was limited by the insufficient coverage of genomic data for extant genera in the Strongylinae and Cyathostominae subfamilies (9/19 genera lacking data). Future studies should prioritize filling the genomic data for key genera (such as *Oesophagodontus* and *Skrjabinodentus*) to enhance the reliability of the topological structure. In addition, the coordinated analysis of morphological trait quantification (such as buccal capsule depth) and molecular evolutionary rates remains unexplored. Subsequent studies can further dissect the mechanisms of species differentiation by integrating multi-omics data from geographical population samples. These expansion directions will systematically improve the classification framework of the Strongylidae family and provide new dimensions for the study of the adaptive evolution of parasitic nematodes.

## Conclusions

In the present study, the complete mitogenome sequence of *P. skrjabini* was assembled, and comparative mitogenomes and phylogenetic analyses with other Strongyloidae species were carried out. Using next-generation sequencing technology, our analyses provided additional molecular evidence to clarify the systematic position of *P. skrjabini*, further improving the understanding of phylogenetic relationships within the Strongylidae family. Our study enriches the database of Strongylidae mitogenomes. The availability of the complete mitogenome of *P. skrjabini* also provides useful genetic markers for molecular epidemiology, population genetics, and systematics of Strongylidae nematodes infecting equids.

## Data Availability

The mitochondrial DNA sequences of *Petrovinema skrjabini* obtained in the present study were deposited in the GenBank database under the BioSample: SAMN17267219; BioProject: PRJNA690898; Sequence Read Archive: SRS7997327; GenBank Accession number: OP784481.

## Abbreviations

ML: Maximum likelihood
BI: Bayesian inference
PCGs: Protein-coding genes
RSCU: The relative synonymous codon usage
GA: Gene arrangement
Ks: Synonymous
Ka: Non-synonymous
GTR: General time-reversible
I: Invariable sites
G: Gamma distribution
Iss: Index of substitution saturation
